# Evolving Molecular Epidemiological Profile of Human Immunodeficiency Virus 1 in the Southwest Border of China

**DOI:** 10.1371/journal.pone.0107578

**Published:** 2014-09-10

**Authors:** Yingyu Chen, Song Chen, Jun Kang, Hua Fang, Hong Dao, Weizhong Guo, Chunhui Lai, Mingyue Lai, Jianhua Fan, Linchun Fu, Jean-Marie Andrieu, Wei Lu

**Affiliations:** 1 Sino-French Collaborative Laboratory, Tropical Medicine Institute, Guangzhou University of Chinese Medicine, Guangzhou, China; 2 Institut de Recherche sur les Vaccins et l’Immunologie des Cancers et du Sida, Université Paris Descartes/Institut de Recherche pour le Développement, Paris, France; 3 Xishuangbanna Center for Disease Control and Prevention, Jinghong, Yunnan, People’s Republic of China; University of Athens, Medical School, Greece

## Abstract

**Background:**

We have previously reported in Xishuangbanna (Banna) Dai Autonomous Prefecture, a well-developed tourist destination in the southwest border of China, that HIV-1 transmitted dominantly through heterosexual contact with less divergent genotypes and few drug resistant mutations [1]. Due to the rapid increase of newly diagnosed HIV-1 cases per year in Banna in recent years, it’s important to evaluate the evolution of HIV-1 molecular epidemiology for the better understanding of ongoing HIV-1 outbreak in this region.

**Methodology/Principal Findings:**

By sequencing of HIV-1 *pol* genes and phylogenetic analysis, we conducted a molecular epidemiologic study in 352 HIV-1-seropositive highly active antiretroviral treatment (HAART)-naïve individuals newly diagnosed at the Banna Center for Disease Control and Prevention between 2009 and 2011. Of 283 samples (84.1% taken from heterosexually acquired adults, 10.6% from needle-sharing drug users, 2.8% from men who have sex with men, 0.4% from children born from HIV-1-infected mothers, and 2.1% remained unknown) with successful sequencing for *pol* gene, we identified 108 (38.2%) HIV-1 subtype CRF08_BC, 101 (35.7%) CRF01_AE, 49 (17.3%) CRF07_BC, 5 (1.8%) C/CRF57_BC, 3 (1.1%) B’, 1 (0.4%) B/CRF51_01B, and 16 (5.7%) unique recombinants forms. Among these infected individuals, 104 (36.7%) cases showed drug resistant or resistance-relevant mutations, and 4 of them conferring high-level resistance to 3TC/FTC, EFV/NVP or NFV. Phylogenetic analysis revealed 21 clusters (2–7 sequences) with only 21.2% (60/283) sequences involved.

**Conclusion/Significance:**

In contrast to our previous findings, CRF08_BC, replaced CRF01_AE, became the dominant genotype of HIV-1 in Banna prefecture. The viral strains with drug resistance mutations were detected frequently in newly diagnosed HIV-1-infected individuals in this region.

## Introduction

The Xishuangbanna (Banna) Dai Autonomous Prefecture of China’s Yunnan province, geographically located at the southwestern border of China, has two counties (Menghai and Mengla) and one city (Jinghong) with an estimated population of 1.13 million populations (including 13 ethnics) in 2012. Due to its distinctive biogeographic characteristics and diversified ethnic cultures, Banna prefecture has become a well-developed tourist destination in China since 1990s.

Before 2005, HIV-1 transmission in Banna prefecture had been characterized as mainly through heterosexual contact (77.3%) with less genotypic diversity and uncommon drug resistance mutations (DRMs) [1]. Although multiple HIV-1 genotypes (including B, B’, C, CRF07_BC, CRF08_BC, and CRF01_AE) had been identified in Yunnan province [2], only CRF01_AE and CRF08_BC had been found to be dominant genotypes (62.2% and 33.3% respectively). Moreover, only one case with subtype CRF01_AE had been identified to carry mutations conferring high-level resistance to antiretroviral drugs NRTI and NNRTI in our previous study [1].

Due to the rapidly increasing numbers of newly diagnosed HIV-1 cases per year in the past years (75 cases in 2005 and nearly 200 cases in 2011), it is important to evaluate the evolution of HIV-1 molecular epidemiology for the better understanding of ongoing HIV-1 outbreak in this region. In addition, the free HAART drugs have been delivered to registered HIV-1-infected individuals with a CD4 count less than 350 cells/µl according to the national “free antiretroviral treatment program” (NFATP) since 2005. Therefore, the transmission of HIV-1 with drug-related mutations might be emerged, as we observed recently in Guangdong province [3]. In the present study, we conducted a molecular epidemiological investigation in 357 cases of newly diagnosed HIV-1 infection from July 2009 to June 2011 at the Banna Center for Disease Control and Prevention (CDC).

## Materials and Methods

### Participants and specimens

From July 2009 to June 2011, a total of 352 individuals who had been newly diagnosed with HIV-1 infection at the Banna CDC had been enrolled into this study. All individuals were required to complete standardized questionnaires (describing sex, age, risk factors, mode of transmission, occupation, geographic location). An enzyme immunoassay for screening HIV-1/HIV-2/O antibodies (Vironostika Uni-form II plus O kit; Organon Teknika BV, Turnhout, Belgium) in sera was performed in the Banna CDC. The positive sera were confirmed by western blot (HIVBlot2.2 kit; Genelabs Diagnostics, Singapore).

After giving their written informed consents, 322 HIV-1 infected individuals were recruited into the molecular epidemiological and drug resistance survey. Briefly, a total of 5 ml of EDTA-treated whole blood was taken from each patient, and plasma samples were sent to our laboratory of Tropical Medicine Institute, Guangzhou University of Chinese Medicine (TMI, GUCM) by flight within 4 hours. The blood samples were used immediately for routine blood count and CD4 T-cell count measurements as well as to separate plasma. Plasma samples were stored at −80°C until use.

### Ethics statement

The institutional ethics committees of TMI, GUCM had approved the study protocol (No. 2009C015).

### Viral load measurement

All plasma samples were thawed at the same time and were used for viral RNA measurement using the Food and Drug Administration (FDA) -approved Amplicor HIV-1 Monitor Test kit (version 1.5) (Roche Molecular Systems, Inc., Branchburg, New Jersey, USA) according to the manufacturer’s instructions.

### Sequencing of HIV-1 *pol* gene

The *pol* gene of HIV-1 encodes the viral enzymes including protease, reverse transcriptase, and integrase, and holds sufficient variability to permit the phylogenetic reconstruction of transmissions [4]. Although it is hard to identify the difference between subtypes CRF01_AE and CRF15_01B by sequencing *pol* region, CRF15_01B so far has not yet been identified in Yunnan province [5] and few cases with CRF15_01B have been reported only in Hebei province and Beijing city [6]. In addition, the newly identified circulating recombinant forms, CRF57_BC [7]/CRF65_cpx [8] and CRF51_01B [9], could not be distinguished from subtypes B and C by sequencing *pol* region. However, considering that subtypes B and C had not-yet been identified in this area, we conducted the HIV-1 genotyping by sequencing the pol region as described previously [1]. Briefly, viral RNA was extracted from the HIV-1-infected individual’s plasma (150 µl) using the QIAamp Viral RNA Mini kit (Qiagen, Valencia, California, United States) according to the manufacturer’s instructions initially. The viral RNA was then subjected to a one-step reverse transcription polymerase chain reaction (RT-PCR) to generate a fragment of *pol* gene (1864 base pairs) spanning protease and reverse transcriptase regions as previously described [1], [3], [10]. The PCR products were purified (Qiagen, Valencia, Spain) and directly sequenced. The sequences generated were edited using the SeqMan II software program from the DNAStar package v.5.08 (Lasergene, Madison, WI). To eliminate potential contamination, all of the sequences obtained were first subjected to an HIV-1 Blast search to compare with related reference sequences in the HIV Databases, funded by the Division of AIDS of the National Institute of Allergy and Infectious Diseases (NIAID), National Institutes of Health (NIH) (http://hiv-web.lanl.gov/content/index). Finally, a total of 283 sequences were successfully obtained from the 322 blood samples.

### Drug resistant mutations

Drug resistance mutations in protease and reverse transcriptase genes were identified by using the last updated online Stanford Resistance Database tool: HIVdb program-Genotypic Resistance Interpretation Algorithm (version 7.0, http://sierra2.stanford.edu/sierra/servlet/JSierra?action=sequenceInput). In additional, sequence data were also submitted and evaluated by using the last updated list for surveillance of transmitted drug-resistant strains in untreated patients (ver.6.0 http://cpr.stanford.edu/cpr.cgi). Resistance mutations identified with HIVdb algorithm were also interpreted referring to the “Update of the drug resistance mutations in HIV-1: March 2013” published by IAS–USA Drug Resistance Mutations Group.

### HIV-1 genotyping and phylogenetic analysis

HIV-1 subtype and CRF designations were determined by uploading sequences into the REGA HIV-1 automated Subtyping Tool version 2.0 (http://www.bioafrica.net/rega- genotype/html/subtypinghiv.html), and confirmed by in-house phylogenetic analysis on nucleotide acid sequences of *pol* as previously described [1], [3], [10]. For phylogenetic analysis, reference sequences representing overall HIV-1 group M genetic variability were obtained from the National Institutes of Health/National Institute of Allergy and Infectious Diseases (NIH/NIAID)-funded HIV database, including all subtypes, sub-subtypes, and circulating recombinant forms (CRFs) references. CRFs identified most recently, such as CRF62_BC [11], CRF64_BC [12], and CRF65_CPX [8], etc., were also included. All reference sequences and our newly obtained nucleotide sequences were aligned using Muscle [13] and followed by manual editing. Neighbor-joining (N-J) tree was drawn under the HKY model of evolution with 1000 bootstrap replicates in SeaView v4.3 [14]. Sequences assigned to specific HIV-1 subtypes were finally confirmed by constructing maximum likelihood (ML) phylogenetic sub-trees as described below. To determine a recombinant virus, similarity analysis and bootscanning were performed with the Simplot version 3.5.1 software [15].

Phylogenetic inter-relationships among viral sequences were estimated using maximum likelihood (ML) phylogenies with PHYML 3.0 [16] with the minimal number of reference sequences. The whole sequence alignment was split into 6 sub-datasets based on the neighbor-joining tree, followed by gap-stripping and ML phylogenetic reconstruction. An approximate likelihood ratio test (SH-like) was used to assess confidence in topology. Phylogenies were inferred using a general-time reversible model of nucleotide substitution, an estimated proportion of invariant sites, and gamma distributed rates among sites. The best of SPR and NNI heuristic options was selected to search the tree space, and bootstrap values with 1000 replicates were used to assess confidence in topology. The existence of transmission clusters was determined using the statistical robustness of the maximum likelihood topologies assessed by high bootstrap values (>98%) with 1000 re-samplings and short branch lengths (genetic distances<0.015) of HIV-1 pol gene sequence [3], [4]. The phylogenetic tree was drawn with FigTree v.1.42 (tree.bio.ed.ac.uk/software/figtree/).

### Statistical analysis

Differences in the CD4 T-cell count and viral load among different subgroups of HIV-1 infected individuals were determined using a Mann–Whitney nonparametric test.

### Accession Numbers

All *pol* sequences analyzed in the present study are deposited in EMBL under the accession numbers HG421451 to HG421735.

## Results

### Characteristics of HIV-1 participants in Banna prefecture

**Figure 1:**
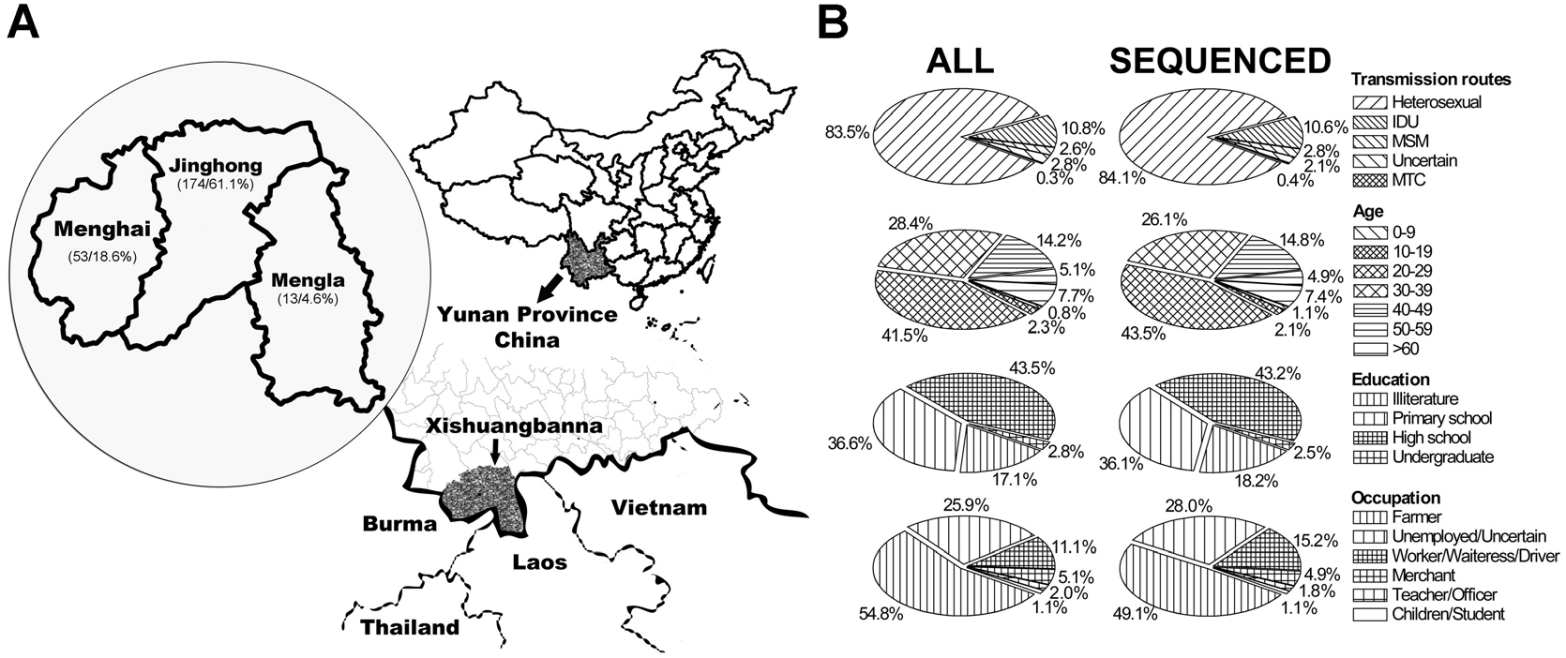
Characteristics of HIV-1 infected individuals in Banna DAI autonomous prefecture in July 2009 to June 2011. The geographical location of Banna prefecture and geographical distribution of infections were presented (parentheses showed cases and percentage of local infected individuals enrolled in this region (A). The characteristics (including their age, occupation, risk factors and education) of all enrolled individuals and individuals with successful sequencing (B).

A total of 352 newly HIV-1 infected individuals had been diagnosed in Banna CDC from July 2009 to June 2011, including 345 natives inhabited in Jinghong city (221 cases, 62.8%), Menghai county (108 cases, 30.7%), Mengla county (16 cases, 4.5%) and immigrants (7 cases, 2.0%) (Figure 1A). The majority of them were men (224 cases, 63.6%), 20–49 years old (296 cases, 84.1%), poorly educated (illiterate or primary school) (155 cases, 44.3%), engaged in farmer (97 cases, 27.6%), unemployment or uncertain (284 cases, 80.7%), and infected mainly by heterosexual contact (294 cases, 83.5%) (Figure 1B). The means (±SD) of CD4 counts and of viral loads were 430±266 cells/ml and 3.76±1.35 log10 copies/ml respectively. All patients had not been previously treated with any antiretroviral drug.

### HIV-1 Genotyping and Unique Recombinant Forms (URF) identification

Among 322 infected individuals who had given their written consent to participate in this study, we obtained the complete sequencing from 283 samples, while 39 samples failed in amplification. However, the characteristics of the 283 individuals with a successful pol sequencing were comparable with that of the whole study group (352 infected individuals) (Figure 1B). Their means (±SD) of CD4 counts and of viral loads were 426±257 cells/ml and 3.84±1.32 log10 copies/ml respectively. Both values were similar to that of the whole study group (see above).

**Figure 2:**
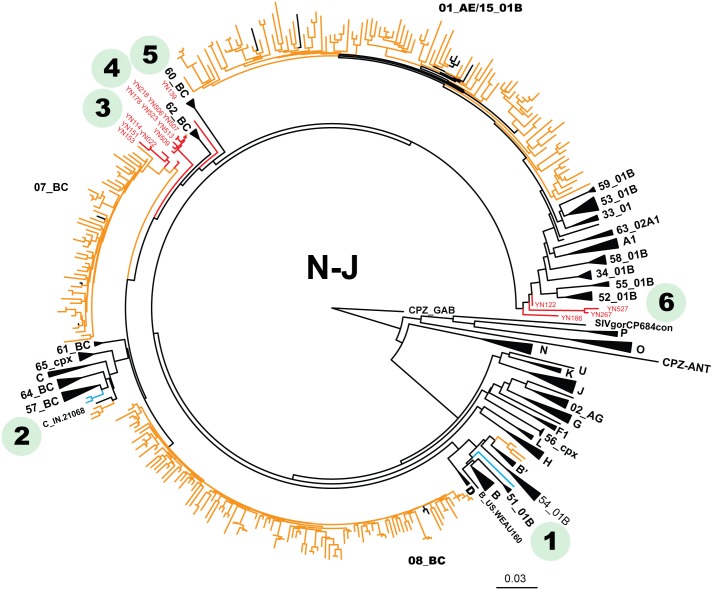
Phylogenetic neighbor-joining tree of HIV *pol* sequences from infected subjects sampled in Banna from 2009 to 2011. The sample sequences with ambiguous genotype were highlighted in blue and red, and labeled with numbers from 1 to 6 simultaneously. **1** and **2** indicated sequences can’t be well distinguished by this sequencing region, and were designated as B/CRF51_01B and C/CRF57_BC respectively. **3** to **6** indicated 16 clustered unique recombinant forms. Other sample sequences and reference sequences were showed in light brown and black respectively.

**Figure 3:**
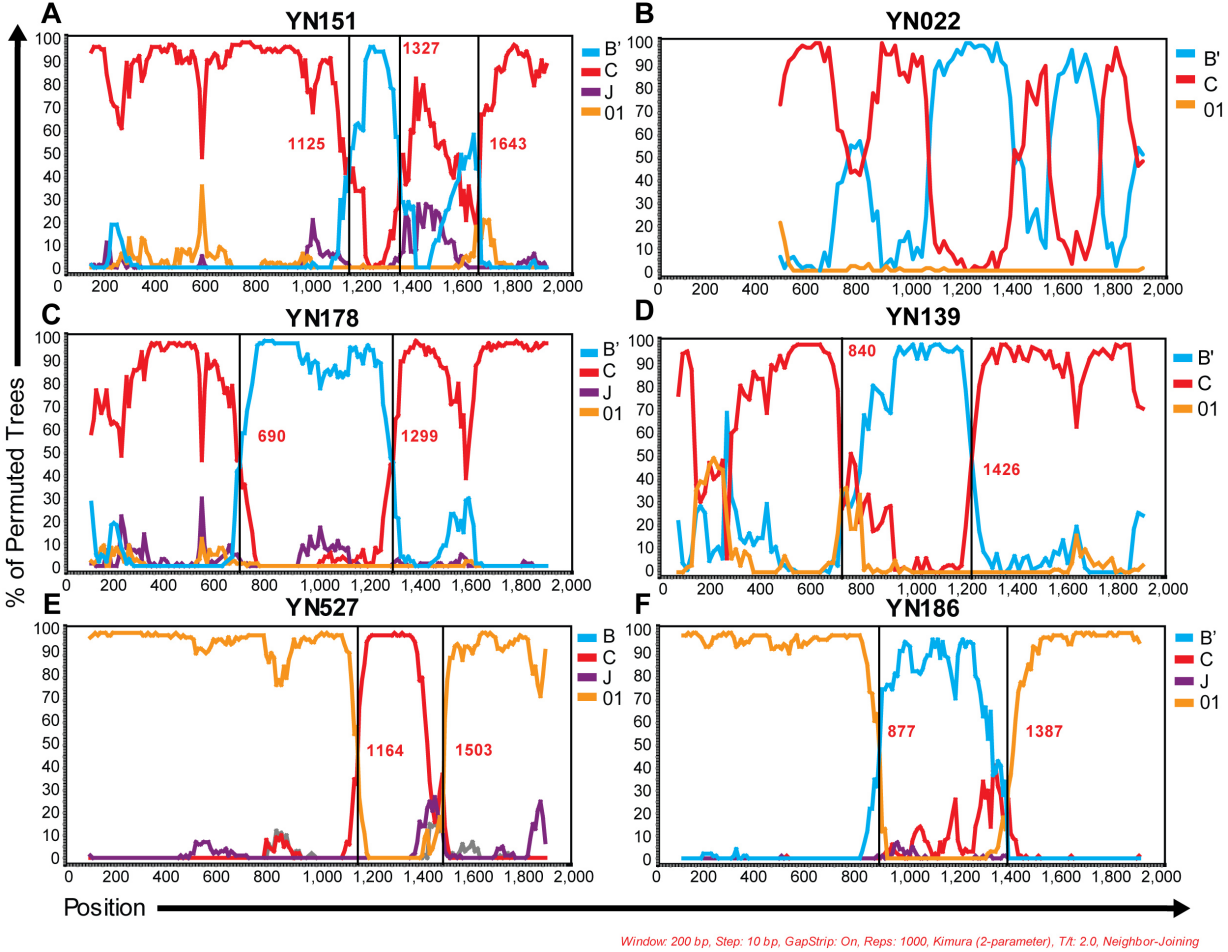
Representative bootscan plots of novel recombinants identified in Banna Prefecture. Simplot bootscan analyses were implemented based on sequences of *pol* region. YN151 (A) and YN022 (B) represented 4 mosaic B’/C recombinants close to CRF07_BC in phylogenetic tree. YN178 (C) and YN139 (D) served as examples of 8 novel B’/C recombinants carried breakpoints similar to those of CRF62_BC. YN527 (E) represented 2 undefined CRF01_AE/C recombinants and YN186 (F) represented 2 undefined CRF01_AE/B’ recombinants.

**Figure 4:**
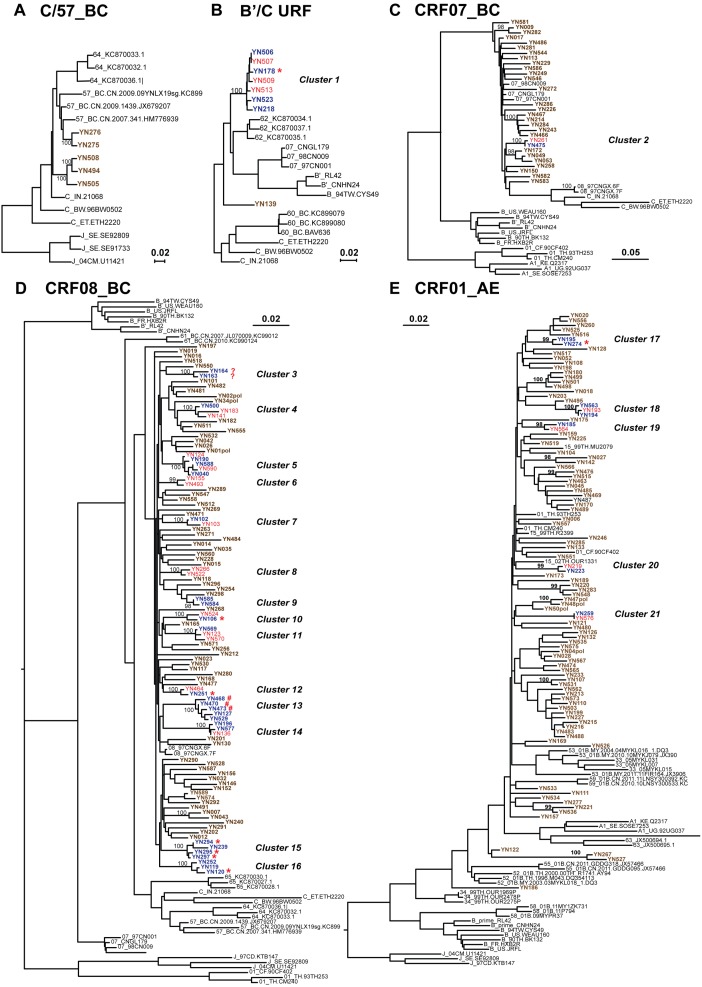
Maximum likelihood tree representing the phylogenetic relationships between HIV-1 infectors in Banna. The whole alignment was split into 6 sub-datasets based on their genotypes, and phylogenetic reconstructions were implemented on each sub-dataset after gag stripping. A total of 21 transmission clusters were identified according to the criteria (bootstrap value>98% and branch lengths<0.015). Sequences composed these clusters were highlighted in blue (male) or red (female), and behind the sequence names, their transmission routes except for heterosexual contact were indicated with asterisk (*, injection drug use), pound sign (#, men who had sex with men) or question mark (?, uncertain) respectively. Unrelated sequences and reference sequences were showed in blown or black respectively.

Of 283 samples with genotyping analysis, 108 (38.2%) CRF_08BC, 101 (35.7%) CRF_01AE, 49 (17.3%) CRF_07BC, 5 (1.8%) C/CRF57_BC, 3 (1.1%) B’, 1 (0.4%) B/CRF51_01B, and 16 (5.7%) URFs had been identified (Table 1 and Figure 2). As showed in Figure 2, sequences with ambiguous genotyping were highlighted in blue and red. Blue lines represented 3 sequences designated as B/CRF51_01B or C/CRF57_BC respectively. Red lines indicated 16 URFs (including 12 B’/C, 2 01_AE/B’ and 2 01_AE/C recombinants). Bootscan analysis revealed that these URFs possessed breakpoints differed apparently from any known reference sequences (Figure 3), even though we included all novel B’/C recombinants circulated in western China (such as CRF62_BC [11], CRF64_BC [12] and CRF65_CPX [8]). As the representative data showed in Figures 3A and 3B, 4 B’/C URF samples (including YN022, YN114, YN151 and YN153) within a cluster of phylogenetic tree, possessed a distinct multiple-mixed B’/C mosaic structure in *pol* region. The other 8 B’/C URFs carried similar structural *pol* region with CRF62_BC. Specifically, they presented a mosaic *pol* structure of C/B’/C with an about 600 bp B’ fragment in the middle (Figures 3C and 3D). Nonetheless, in our sequence alignment, the breakpoints of CRF62_BC located at positions 1034 and 1610, about 200 bp or 300 bp behind than breakpoints of YN139 or YN178 respectively. Similarly, the remaining URFs (2 01_AE/B’ and 2 CRF01_AE/C) also could not be defined as any known CRF based on their bootscan analysis (Figure 3E and 3F). Full genome sequencing is required for confirming any novel circulating recombinant form in these samples.

**Table 1:** HIV-1 genotypes and transmission routes of infected individuals in Banna prefecture, China (cases, percentage).

	Transmission Routes											
Genotypes	Heterosexual		MSM		IDU		MTC		Uncertain		Total	
CRF08_BC	88	81.5%	1	0.9%	16	14.8%	1	0.9%	2	1.9%	108	38.2%
CRF01_AE	94	93.1%	3	3.0%	4	4.0%					101	35.7%
CRF07_BC	32	65.3%	3	37.5%	11	35.5%			3	60.0%	49	17.3%
C/CRF57_BC	5	100.0%									5	1.8%
B’	3	100.0%									3	1.1%
B/CRF51_01B	1	100.0%									1	0.4%
**URF** *												
B’/C URF	11	91.7%	1	8.3%							12	4.2%
CRF01_AE/B’	2	100.0%									2	0.7%
CRF01_AE/C	2	100.0%									2	0.7%
**Total**	**238**	**84.1%**	**8**	**2.8%**	**31**	**11.0%**	**1**	**0.4%**	**5**	**1.8%**	**283**	**100.0%**

*Recombinants were determined by bootscan analysis with Simplot software.

MSM, men who have sex with men; MTC, mother to child transmission; IDUs, intravenous drug users; URF, unique recombinant forms.

### Drug Resistance Mutation Analysis (DRMs)

As showed in Table 2, a total of 33 DRMs sites in 104/283 (36.7%) infected individuals were identified with HIVdb Algorithm in our study, including 6 DRMs identified by CPR ver.6.0 simultaneously. The 6 DRMs identified by both HIVdb Algorithm and CPR included M184V/I, T215S, K219N (NRTI), K103N (NNRTI), and D30N, M46I/L (PI). M184V is selected by 3TC/FTC and reduces susceptibility to these drugs more than 100-fold. M184I usually emerges before M184V and has similar resistance profiles. T215S is a known revertant of the resistance mutation T215Y/F. K103N is selected by NVP and EFV and reduces susceptibility to them by about 50 and 20-fold, respectively. D30N is a NFV-selected substrate-cleft mutation that causes high-level resistance to NFV. M46I/L are selected primarily by NFV, ATV and LPV etc., and reduce susceptibility to these drugs. Other mutations such as A98G, V179D/T and Q58E are also need to be considered. A98G and V179D reduces NVP and EFV susceptibility. Q58E is a major mutation for Tipranavir.

**Table 2:** Drug resistant mutations (DRM) identified in HAART-naïve newly diagnosed infected individuals in Banna, China.

Category	DRM	Cases	Suspectivity						
**NRTI**			**3TC**	**ABC**	**AZT**	**D4T**	**DDI**	**FTC**	**TDF**
	A62V	1	**S**	**S**	**S**	**S**	**S**	**S**	**S**
	T69N	5	**S**	**S**	**S**	**S**	**PL**	**S**	**S**
	T69S	7	**S**	**S**	**S**	**S**	**S**	**S**	**S**
	**M184V**	1	**H**	**L**	**S**	**S**	**PL**	**H**	**S**
	**M184I**	1	**H**	**L**	**S**	**S**	**PL**	**H**	**S**
	**T215S**	1	**S**	**PL**	**L**	**L**	**PL**	**S**	**S**
	**T215A**	1	**S**	**PL**	**L**	**L**	**PL**	**S**	**S**
	***K219N***	1	**S**	**S**	**PL**	**PL**	**S**	**S**	**S**
**NNRTI**			**EFV**	**ETR**	**NVP**	**RPV**			
	V90I	17	**S**	**S**	**S**	**S**	**S**	**S**	**S**
	A98G	8	**PL**	**PL**	IM	**L**			
	*L100V*	1	IM	**PL**	IM	**L**			
	**K103N**	1	**H**	**S**	**H**	**S**			
	*K103E*	1	**S**	**S**	**S**	**S**			
	V106I	4	**S**	**S**	**S**	**S**			
	E138A	13	**S**	**PL**	**S**	**L**			
	V179D	17	**PL**	**PL**	**PL**	**PL**			
	V179T	7	**PL**	**PL**	**PL**	**PL**			
	*G190R*	1	**S**	**S**	**S**	**S**			
	H221Y	1	**PL**	**PL**	**PL**	**PL**			
**PI Major**			**ATV/r**	**DRV/r**	**FPV/r**	**IDV/r**	**LPV/r**	**NFV**	**SQV/r**
	**D30N**	**1**	**S**	**S**	**S**	**S**	**S**	**H**	**S**
	**M46I**	**1**	**PL**	**S**	**PL**	**PL**	**PL**	**L**	**S**
	**M46L**	**2**	**PL**	**S**	**PL**	**PL**	**PL**	**L**	**S**
**PI minor** *									
	L10V	8	**S**	**S**	**S**	**S**	**S**	**S**	**S**
	L10I	8	**S**	**S**	**S**	**S**	**S**	**S**	**S**
	L10F	1	**S**	**S**	**PL**	**PL**	**S**	**PL**	**S**
	V11I	2	**S**	**S**	**S**	**S**	**S**	**S**	**S**
	K20I	1	**S**	**S**	**S**	**S**	**S**	**PL**	**S**
	L33I	1	**S**	**S**	**S**	**S**	**S**	**S**	**S**
	*G48R*	1	**S**	**S**	**S**	**S**	**S**	**S**	**S**
	Q58E	3	**S**	**S**	**S**	**S**	**S**	**L**	**S**
	A71T	3	**S**	**S**	**S**	**S**	**S**	**S**	**S**
	A71V	6	**S**	**S**	**S**	**S**	**S**	**S**	**S**
	*T74S*	3	**S**	**S**	**S**	**S**	**S**	**L**	**S**
**TOTAL 33 DRMs 130 (in 104/283 infectors, 36.7%)**									

Note: DRMs analyses were implemented based on HIVdb algorithm. Bold font indicated DRMs which were also included in CPR ver.6.0 (SDRM 2009). Italic font indicated DRMs not included in 2013 IAS-USA.

*L33F is polymorphic in 19 individuals infected with HIV-1 subtype CRF01_AE.

NRTI, nucleoside reverse transcriptase inhibitors; NNRTI, non-nucleoside reverse transcriptase inhibitors; PI, protease inhibitors; 3TC, lamivudine; FTC, emtricitabine; ABC, abacavir; AZT, zidovudine; D4T, stavudine; DDI, didanosine; EFV, efavirenz; NVP, nevirapine; NFV, nelfinavir; ATV, Atazanavir; LPV/r, lopinavir and ritonavir; IDV, indinavir; S, susceptible; H, high-level resistance; I, intermediate resistance; L, low-level resistance; and PL, potential low-level resistance.

### Identification of Transmission Clusters

To evaluate the global profile of the local HIV-1 transmission, we split the sequence alignment into 6 sub-datasets, and implemented maximum-likelihood phylogenetic reconstruction on each sub-dataset to obtain the genetic distances. These sub-datasets were designate with the data of a representative references, such as CRF08_BC (1467 base pairs remained after gap stripping), 07_BC (1666 base pairs), B/CRF51 (1711 base pairs), CRF01_AE (1194 base pairs), C/CRF57 (1752 base pairs) and B’/C URF (1708 base pairs). As screened by our criteria (bootstrap value>98% and branch lengths<0.015), 21.2% (60/283) of all sequenced samples analyzed segregated into 21 transmission clusters (Figure 4). Of those 21 transmission clusters identified, 17 chains had been supported by contact tracing (including the sex, age, habitation, occupation, risk infectors in the same cluster), while four clusters (clusters 3, 6, 8 and 9) remained ambiguous. Clusters 6 and 8 were both consisted of 2 women lived in the same city, who had reported to get HIV-1 from heterosexual contact. Both clusters 3 and 9 derived from 2 men lived in the same city, who claimed to contract HIV-1 either by heterosexual contact or by uncertain route.

Among the 21 clusters, most of them were composed of 2 sequences (12 clusters), while 5 clusters were composed of 3 sequences and 4 clusters were composed of multiple sequences (cluster 15 consisted of 4 sequences; both clusters 5 and cluster 13 consisted of 5 sequences; and the biggest cluster 1, consisted of 7 sequences). The new B’/C recombinant virus spreading in cluster 1 represented a potential regional outbreak of novel B’/C recombinant, as reported elsewhere in Yunnan province [17], [18]. The characteristics of patients in the 21 transmission clusters were summarized in Table 3.

**Table 3:** Characteristics of infectors in 21 transmission clusters identified by phylogenetic reconstruction based on *pol* sequences.

Cluster No.	Patient No.	Risk Factors	Sex	Age	Location	Genotype	CD4	PVL	Drug resistant mutations (HIVdb)			
									PI Major	PI Minor	NRTI	NNRTI
1	YN178	IDU	Male	76	Jinghong	B’/C URF	341	7526				
	YN218	Heterosexual	Male	76	Jinghong	B’/C URF	564	208505				
	YN506	Heterosexual	Male	66	Jinghong	B’/C URF	339	12951				
	YN507	Heterosexual	Female	64	Jinghong	B’/C URF	490	1870				
	YN509	Heterosexual	Female	68	Jinghong	B’/C URF	479	140719				
	YN513	Heterosexual	Female	64	Jinghong	B’/C URF	432	12900				
	YN523	Heterosexual	Male	80	Jinghong	B’/C URF	206	10530				
2	YN261	Heterosexual	Female	48	Jinghong	CRF07_BC	254	20820				
	YN475	Heterosexual	Male	47	Jinghong	CRF07_BC	135	128310				
3	YN163	Uncertain	Male	27	Jinghong	CRF08_BC	967	5450				
	YN164	Uncertain	Male	31	Jinghong	CRF08_BC	191	100560				
4	YN141	Heterosexual	Female	62	Jinghong	CRF08_BC	651	5563				V179T
	YN183	Heterosexual	Female	61	Jinghong	CRF08_BC	176	12525				V179T
	YN500	Heterosexual	Male	76	Jinghong	CRF08_BC	168	19633				V106IV, V179T
5	YN040	Heterosexual	Male	27	Menghai	CRF08_BC	249	156805				V90I
	YN124	Heterosexual	Female	49	Menghai	CRF08_BC	190	44594				V90I
	YN190	Heterosexual	Male	38	Menghai	CRF08_BC	273	1050				V90I
	YN588	Heterosexual	Male	32	Menghai	CRF08_BC	319	32013				V90I
	YN590	Heterosexual	Female	24	Menghai	CRF08_BC	564	108756				V90I
6	YN155	Heterosexual	Female	41	Jinghong	CRF08_BC	444	324				
	YN493	Heterosexual	Female	33	Jinghong	CRF08_BC	579	167500				
7	YN102	Heterosexual	Male	39	Jinghong	CRF08_BC	191	4108				E138A
	YN103	Heterosexual	Female	27	Jinghong	CRF08_BC	454	654043				
8	YN266	Heterosexual	Female	28	Jinghong	CRF08_BC	440	1527400				
	YN522	Heterosexual	Female	25	Jinghong	CRF08_BC	840	2579				
9	YN584	Heterosexual	Male	28	Puer	CRF08_BC	521	<50				E138A
	YN585	Heterosexual	Male	29	Puer	CRF08_BC	461	<50				E138A
10	YN106	IDU	Male	48	Jinghong	CRF08_BC	53	1665004				
	YN524	Heterosexual	Female	35	Mengla	CRF08_BC	1178	1320				
11	YN123	Heterosexual	Female	23	Menghai	CRF08_BC	400	2095				
	YN569	Heterosexual	Male	25	Menghai	CRF08_BC	392	3328				
	YN570	Heterosexual	Female	23	Menghai	CRF08_BC	139	4114				
12	YN251	IDU	Male	28	Jinghong	CRF08_BC	462	1871				
	YN464	Heterosexual	Female	16	Jinghong	CRF08_BC	495	25533				
13	YN127	Heterosexual	Male	28	Jinghong	CRF08_BC	1073	16700				
	YN468	MSM	Male	25	Jinghong	CRF08_BC	515	2301				
	YN470	MSM	Male	38	Jinghong	CRF08_BC	387	40423				
	YN473	MSM	Male	25	Jinghong	CRF08_BC	350	38508				
	YN529	Heterosexual	Male	36	Jinghong	CRF08_BC	623	16965				
14	YN136	Heterosexual	Female	20	Menghai	CRF08_BC	396	1068		Q58E, T74S		
	YN196	Heterosexual	Male	29	Menghai	CRF08_BC	387	1370		Q58E, T74S		
	YN577	Heterosexual	Male	29	Menghai	CRF08_BC	594	17220		Q58E, T74S		
15	YN239	Heterosexual	Male	28	Jinghong	CRF08_BC	355	738				V90I, V179D
	YN295	IDU	Male	31	Jinghong	CRF08_BC	523	1204				V90I, V179D
	YN297	IDU	Male	33	Jinghong	CRF08_BC	619	1109				V90I, V179D
	YN294	IDU	Male	30	Jinghong	CRF08_BC	604	779				V90I, V179D
16	YN119	Heterosexual	Male	19	Jinghong	CRF08_BC	309	131000				
	YN120	IDU	Male	23	Jinghong	CRF08_BC	351	790690				Y181HY
	YN252	Heterosexual	Male	42	Jinghong	CRF08_BC	337	1202				
17	YN195	Heterosexual	Male	29	Menghai	CRF01_AE	40	207798		L33F*		
	YN274	IDU	Male	23	Jinghong	CRF01_AE	1618	318		L33F*		
18	YN193	Heterosexual	Female	24	Menghai	CRF01_AE	58	213587				A98G
	YN194	Heterosexual	Male	26	Menghai	CRF01_AE	1042	56				A98G
	YN563	Heterosexual	Male	35	Menghai	CRF01_AE	36	6241				A98G
19	YN185	Heterosexual	Male	59	Jinghong	CRF01_AE	86	84786		L33F*		
	YN564	Heterosexual	Female	26	Menghai	CRF01_AE	195	132000		L33F*		
20	YN219	Heterosexual	Female	35	Jinghong	CRF01_AE	652	2450				
	YN223	Heterosexual	Male	36	Jinghong	CRF01_AE	138	10400				
21	YN259	Heterosexual	Male	26	Menghai	CRF01_AE	480	258460				
	YN576	Heterosexual	Female	22	Menghai	CRF01_AE	711	28202				

We further investigated whether the transmission of drug resistant strains occurs among these 21 clusters. There were 6/21 (28.6%) clusters consisted of individuals who were infected with strains carrying the same DRMs. These cases included V179T in cluster 4, V90I in cluster 5, E138A in cluster 9, Q58E/T74S in cluster 14, V90I/V179D in cluster 15, and A98G in cluster 18.

## Discussion

In Yunnan province, there were a total of 104981 cases of HIV-1 infection and 7671 newly diagnosed HIV-1 infected individuals in 2012 (http://yn.yunnan.cn/html/2012-11/27/content_2510358.htm). Although nearly 200 newly diagnosed HIV-1 infected individuals per year in Banna prefecture represents only 2% of new infections per year in Yunnan, our findings in the present study did raise serious concerns on the severity of regional HIV-1 transmission. Firstly, newly diagnosed infected individuals increased rapidly (averaged 18% per year deduced from cases in 2005 and 2011). Heterosexual transmission was consistently dominant in Banna. In addition, the unsafe sexual behavior of injecting drug users could further increased the risk of HIV-1 transmission in the general population [19].

Secondly, our results suggested that HIV-1 population evolved rapidly in Banna and the evidence of new sources of infection further increased the genetic complexity. The profiles of HIV-1 evolution in Yunnan province (including the emergence of numerous novel circulating recombinant forms) had been described by previous reports [5], [20], [21], [22]. Compared with our previous study in 2008 [1], CRF08_BC had replaced CRF01_AE to become the most prevalent genotype in this region. A shift of dominant HIV-1 subtype had also been described in other regions of Yunnan province [5], [20], [21]. Moreover, we identified in the present study 6 common genotypes and 3 URF genotypes (undefined B’/C, CRF01_AE/C, and CRF01_B’), as compared to 3 common genotypes and only 1 URF reported in our previous study in the same region [1]. These results indicated the local HIV-1 genotypes divergence did increase rapidly in this region.

A series of novel circulating recombinant forms have recently been identified in China, especially in Yunnan province, including CRF55_01B (identified from MSM in China) [23], CRF57_BC [7], CRF59_01B (from MSM in northeast China) [24], CRF61_BC (CRF found among the heterosexual population in different regions in China) [25], CRF62_BC [11], CRF64_BC [12], CRF65_CPX (first novel HIV-1 second-generation inter-CRF in China) [8]. In the present study, we included available sequences of CRFs in HIV-1 sequence database into our reference pool. The breakpoints of our URFs differed apparently from those of any known CRF, implying the unidentified novel CRFs emerged in this region.

The evolving profile of HIV-1 molecular epidemiology raises also a challenge for the antiretroviral therapy. The diagnosis of DRMs associated with free HAART drugs (supplied under the NFATP program) had been increased rapidly in the past 5 years. The rapid genotyping evolution and transmission of HIV-1 with DRMs in Banna might increase the risk of treatment failure in this region. Of 283 sequences analysed, 102 (36.0%) showed DRMs, even if we excluded 6 DRMs from the IAS-USA 2013 mutation list (due to the coexistence of multiple DRMs). This proportion of untreated HIV-1 patients with DRMs was apparently higher than those reported previously (13.3%) [1]. Moreover, not only the rapidly increasing prevalence of resistant strains, but also the emerging of mutations conferring high level resistance to the primary antiretroviral drugs represent a serious challenge for the control of epidemic in this region. As in this study, there were 4 (1.4%) cases carrying mutations M184V (2 cases), K103N (1 case) and D30N (1case), which should confer high level resistance to 3TC/FTC, EFV/NVP and NFV, the primary drugs composed the first-line antiretroviral therapy regimen recommended in China. Moreover, a higher proportion (28.6%) of transmission clusters were also found to be associated with DRMs transmission. Poor adherence and compliance may be the most important contributors related to the increasing prevalence of DRMs in this region. The “HIV-1 Education and Training Programs” need to be enhanced in Banna to guarantee the infected individuals taking medicine as advised. Since as high as 54.8% of infected individuals were farmer and 25.9% were unemployed (Figure. 1b), such a poor education status would frustrate our efforts on prevention and control of HIV-1 dissemination. Thus, the efforts to include people in low social-status into the NFATP program will be the key to control HIV-1 outbreak in this region.
